# Causal Effects of Oral Microbiome, Circulating Metabolites, and Inflammatory Proteins on Recurrent Aphthous Stomatitis: A Two-Sample Mendelian Randomization Study

**DOI:** 10.3290/j.ohpd.c_2774

**Published:** 2026-08-26

**Authors:** Shaobin Long, Wei Zeng, Simin Huang, Lixian Yang, Rong Zhou, Keke Zhu, Xuan Zhu

**Affiliations:** a Shaobin Long Undergraduate Student, Hunan University of Traditional Chinese Medicine, Changsha, Hunan, China. Conceived and designed the study, conducted the analysis, wrote the paper, approved the submitted version.; b Wei Zeng Undergraduate Student, Hunan University of Traditional Chinese Medicine, Changsha, Hunan, China. Conceived and designed the study, approved the submitted version.; c Simian Huang Undergraduate Student, Hunan University of Traditional Chinese Medicine, Changsha, Hunan, China; 3.Department of Stomatology, The First Affiliated Hospital of Hunan, University of Chinese Medicine, Changsha, Hunan, China. Conceived and designed the study, approved the submitted version.; d Lixian Yang Undergraduate Student, Hunan University of Traditional Chinese Medicine, Changsha, Hunan, China. Conducted the analysis, wrote the paper, approved the submitted version.; e Rong Zhou Associate Chief Physician, Changsha Hospital for Maternal and Child Health Care, Changsha, Hunan, China. Donducted the analysis, wrote the paper, approved the submitted version.; f Keke Zhu Associate Chief Physician, Department of Stomatology, The First Affiliated Hospital of Hunan, University of Chinese Medicine, Changsha, Hunan, China. Conceived and designed the study, conducted the analysis, wrote the paper, approved the submitted version.; g Xuan Zhu Lecturer, Hunan University of Traditional Chinese Medicine, Changsha, Hunan, China; Department of Stomatology, The First Affiliated Hospital of Hunan, University of Chinese Medicine, Changsha, Hunan, China. Conceived and designed the study, conducted the analysis, wrote the paper, approved the submitted version.

**Keywords:** blood metabolome, inflammatory proteins, Mendelian randomization, oral microbiota, recurrent aphthous stomatitis.

## Abstract

**Purpose:**

Recurrent aphthous stomatitis (RAS) affects 20% of the global population with unclear etiology and limited treatment options. While observational studies suggest associations with oral microbiome dysbiosis and metabolic alterations, causal relationships have not been established. Using Mendelian randomization, the aim of this study was threefold: 1. to systematically investigate the causal architecture underlying RAS; 2. to partition the total effect of identified microbial taxa into direct and mediator-adjusted components; 3. to determine whether specific circulating metabolites and circulating inflammatory proteins mediate the microbiome–RAS relationship.

**Materials and Methods:**

We conducted two-sample Mendelian randomization (MR) using oral microbiome, metabolomics, inflammatory proteins, and RAS genome-wide association study summary statistics (GWAS). Univariable MR assessed direct causal effects, while multivariable MR evaluated mediation pathways. Sensitivity analyses included MR-Egger, weighted median, and MR-PRESSO.

**Results:**

The order Clostridiales demonstrated protective effects against RAS (OR=0.851, 95% CI: 0.759-0.954, p=0.006). Among 36 causally associated metabolites, myo-inositol (OR=1.619), sphingosine 1-phosphate (OR=1.672), and hexadecadienoate (OR=1.825) increased RAS risk, while 4-acetylphenol sulfate (OR=0.606) and acetylcarnitine (OR=0.604) conferred protection. No inflammatory proteins showed statistically significant associations. Two-step mediation analysis identified sphingomyelin (d18:1/22:1, d18:2/22:0, d16:1/24:1) as a metabolic intermediate in the Clostridiales–RAS pathway, with the direct protective effect of Clostridiales remaining statistically significant after adjustment for sphingomyelin levels.

**Conclusions:**

This study provides genetic evidence supporting causal relationships linking the oral microbiome and metabolites to RAS, and identified Clostridiales as protective against RAS, but found multiple lipid mediators as risk factors. The interplay between Clostridiales and sphingolipid metabolism provides candidate therapeutic targets for RAS management through microbiome modulation or metabolic intervention.

Recurrent aphthous stomatitis (RAS) is one of the most prevalent oral mucosal disorders, affecting approximately 20% of the global population and characterized by recurrent, painful ulcerations that substantially impair quality of life.^47,48^ RAS is recognized as a multifactorial T cell-mediated immune dysregulatory disease, with pathogenesis involving genetic susceptibility, microbial infections, nutritional deficiencies, hormonal imbalances, and psychological stress.^6,12^ Despite extensive investigation, the precise pathogenetic mechanisms remain unclear; no single causative pathway has been established, and systematic reviews demonstrate that no standardized curative treatment currently exists.^3,38^ This etiological complexity underscores the urgent need for mechanistic insights capable of informing targeted therapeutic strategies.

Recent advances in high-throughput omics technologies have substantially expanded our understanding of RAS-associated molecular alterations. Microbiome studies using 16S rRNA gene sequencing have revealed significant microbial dysbiosis at ulcer sites, including decreased *Streptococcus salivarius* and increased *Acinetobacter johnsonii*, with a dysbiosis index predicting 83% of cases.^17,23^ Proteomic and metabolomic analyses have identified differentially expressed proteins and metabolic pathway perturbations — including alterations in vitamin B9/B12, nitrogen, and selenium metabolism — during active ulceration.^7,16,55^ Genetic studies, including genome-wide association studies (GWAS), have identified susceptibility loci at IL12A, STAT4, IL10, and CCR1–CCR3, while rare variant analyses have revealed STAT1 gain-of-function and TNFAIP3 mutations in early-onset pediatric cases.^13,31^. Furthermore, oral microbial diversity correlates inversely with mucosal inflammation severity and pro-inflammatory cytokine levels, supporting the oral microbiome as a potential biomarker for disease activity.^18^ However, these findings are derived from observational study designs that cannot establish whether the observed associations represent true causal relationships. Whether microbiome dysbiosis causes or results from RAS, and whether protein or metabolite alterations are pathogenic factors or compensatory responses, remain fundamental unanswered questions.

Mendelian randomization (MR) addresses these limitations by using genetic variants as instrumental variables for exposures, thereby minimizing bias from confounding and reverse causation inherent in observational designs.^41,44^ MR has been successfully applied to establish causal relationships in autoimmune and inflammatory diseases, such as the causal role of *Bifidobacterium* abundance in type 1 diabetes and celiac disease susceptibility.^51^ Yet no study has systematically employed MR to evaluate causal relationships across the oral microbiome–metabolism–inflammation axis in RAS, leaving a critical gap in our understanding of disease etiology.

The present study employed a three-layer MR framework to systematically investigate the causal architecture underlying RAS. First, univariable MR (UVMR) was used to identify oral microbial taxa with putatively causal effects on RAS and to assess potential reverse causality through bidirectional analysis. Second, multivariable MR (MVMR) was applied to partition the total effect of identified microbial taxa into direct and mediator-adjusted components. Third, two-step mediation was conducted MR to determine whether specific circulating metabolites and circulating inflammatory proteins mediate the microbiome–RAS relationship, thereby delineating the oral microbiome–metabolism–inflammation axis underlying RAS susceptibility.

## MATERIALS AND METHODS

This study used GWAS data from prior research. Ethical approval and consent were obtained in the original studies.

The datasets analyzed during the current study are publicly available. Oral microbiome GWAS summary statistics are available from Stankevic et al.^46^ RAS GWAS data are available from the FinnGen R11 database (https://r11.finngen.fi/). Metabolomics GWAS summary statistics are available from Chen et al.^5^ Inflammatory protein GWAS data are available from Zhao et al.^53^ Summary-level data can also be accessed through the IEU OpenGWAS platform (https://gwas.mrcieu.ac.uk/).

### Study Dand MR Framework

MR employs genetic variants as instrumental variables (IVs) to derive less biased causal estimates through random allele allocation at conception, addressing challenges from confounding and reverse causation that affect observational investigations examining associations between the oral microbiome and complex diseases such as RAS. This investigation adheres to STROBE-MR reporting guidelines (supplementary Table A1 https://www.quintessence-publishing.com/quintessenz/journals/articles/downloads/ohpd_2026_8353_long_appendix.xlsx) and evaluates three core MR assumptions: (1) relevance: IVs demonstrate strong associations with exposures; (2) independence: IVs remain independent of exposure-outcome confounders; (3) exclusion restriction: IVs influence outcomes exclusively through exposures, without alternative pathways.

A comprehensive MR framework was constructed utilizing GWAS summary data to systematically explore causal relationships between oral microbial communities and RAS, examining potential mediation through circulating metabolites and inflammatory proteins. Initial analyses employed UVMR to quantify direct causal effects of oral microbiome taxa on RAS, with reverse-direction UVMR performed to assess reverse causality. Subsequently, UVMR examined oral microbiome effects on circulating metabolites/inflammatory proteins, followed by testing whether these circulating traits causally influenced RAS risk, identifying putative mediators meeting both criteria. MVMR was applied to delineate and quantify independent mediator roles in microbiome-to-RAS pathways and estimate indirect effects.

### Data Sources

Four publicly available large-scale GWAS summary datasets were integrated, encompassing exposures (oral microbiome), putative mediators (circulating metabolites and inflammatory proteins), and outcomes (RAS). Oral microbiome GWAS summary statistics originated from 610 Danish participants, providing genetic associations for 43 oral microbial taxa.^46^ RAS GWAS data were derived from the FinnGen R11 database, including 4678 cases and 449,055 controls. Metabolomics GWAS summary statistics came from the Canadian Longitudinal Study on Aging, encompassing 51,338 individuals and approximately 1400 metabolites, including amino acids, carbohydrates, lipids, nucleotides, and related classes.^5^ GWAS data for 91 inflammatory proteins were obtained from 14,824 individuals of European ancestry.^53^ Participants were predominantly of European descent with minimal sample overlap, supporting sample independence assumptions in two-sample MR.

### Genetic Instrument Selection

Stringent selection criteria ensured IV validity. Considering limited sample sizes for certain GWAS, a suggestive significance threshold of p<5 × 10-5 was applied for initial SNP screening, balancing IV strength against instrument count for adequate power4. LD clumping was performed using 1000 Genomes Project Phase 3 European reference panel with 10,000-kb windows and r² = 0.001, ensuring approximate independence. Addressing weak-instrument bias, F-statistics were calculated for each SNP (F = (β/SE)²), retaining only SNPs with F ≥ 1034.

### Univariable MR Analysis

UVMR was performed to estimate causal effects of each exposure on outcomes using selected IVs. Three complementary estimators were employed via the TwoSampleMR R package (version 0.6.7; R version 4.4.1) to address horizontal pleiotropy: inverse variance weighted (IVW) as primary analysis, efficient in the absence of pleiotropy or under balanced pleiotropy; weighted median, maintaining consistency when up to 50% of IVs are invalid; and MR-Egger, testing and adjusting for directional pleiotropy with reduced power. Circulating metabolites or inflammatory proteins showing statistically significant causal associations with RAS were identified as candidate mediators for subsequent analyses.

### Multivariable MR (MVMR) and Mediation Analysis

MVMR evaluated candidate mediators’ roles in microbiome-to-RAS pathways, estimating direct effects of multiple exposures (microbial taxon and mediator) on RAS while accounting for genetic correlations between exposures. MVMR separated direct microbial taxon effects (independent of mediators) from mediator effects on RAS (adjusted for taxa). Indirect effects were quantified using product-of-coefficients methodology, multiplying microbiota-to-mediator effects from UVMR (β1) by mediator-to-RAS effects from MVMR (β2). The proportion mediated was calculated as (β1 × β2)/β3, where β3 represented total microbiota-to-RAS effects from UVMR. MVMR provided deeper causal inference for dissecting mechanistic pathways and evaluating the importance of identified mediators.

### Sensitivity Analyses

Multiple validation layers for MR assumptions enhanced the credibility of our findings. Cochran’s Q assessed heterogeneity among IVs (with p<0.05 indicating heterogeneity). Horizontal pleiotropy evaluation employed the MR-Egger intercept (p<0.05 suggesting directional pleiotropy) and the MR-PRESSO global test (version 1.0), detecting outliers and providing outlier-corrected estimates (p<0.05 indicating significant pleiotropy). When MR-PRESSO identified outliers, analyses were repeated following their removal. These prespecified sensitivity analyses evaluated core MR assumptions and enhanced robustness.

## RESULTS

### Oral Microbiome-RAS Causal Relationships

**Fig 1:**
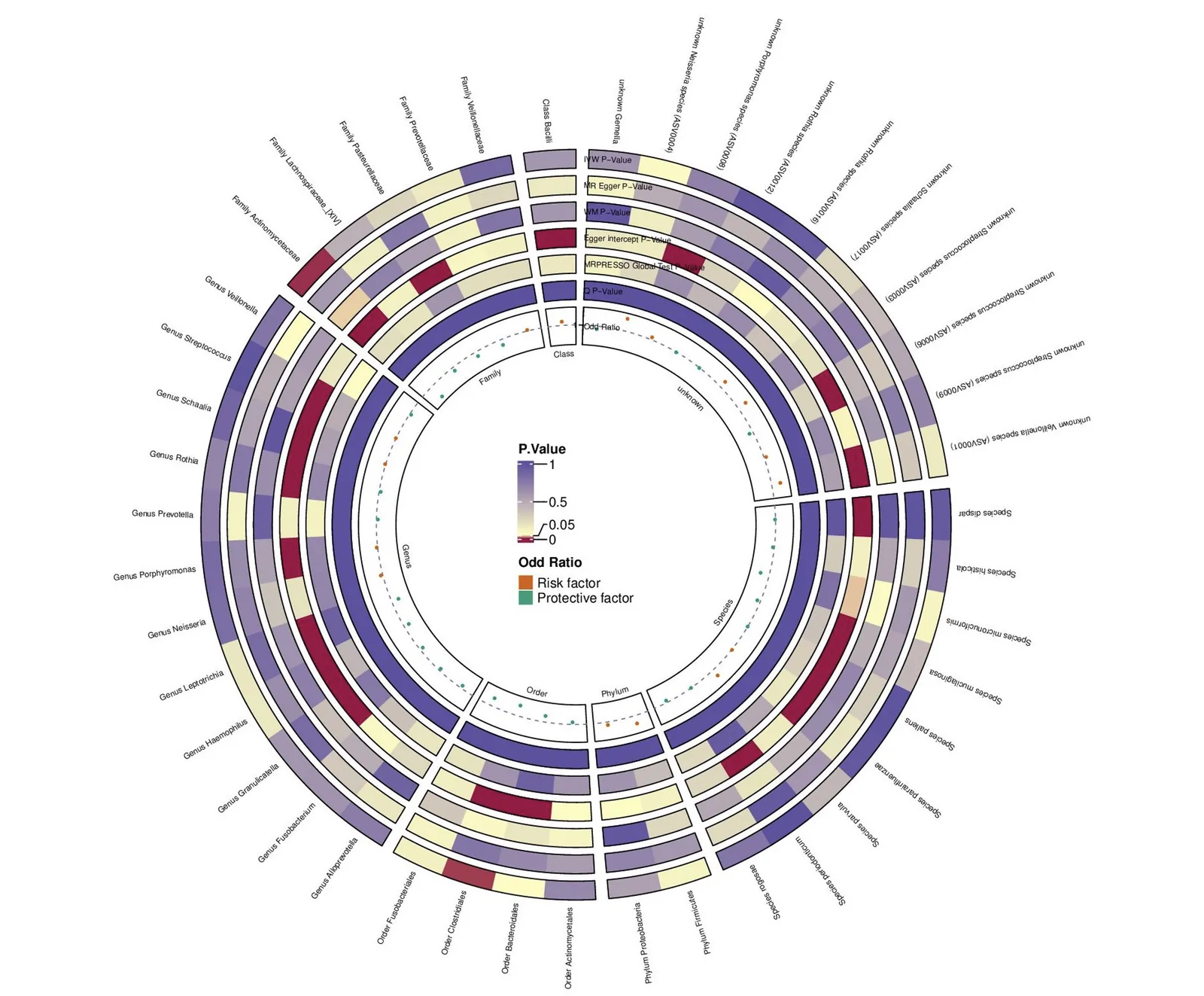
Causal associations between oral microbiota and RAS using two-sample Mendelian randomization. Circular heatmap summarizing the Mendelian randomization results between 43 oral microbial taxa and recurrent aphthous stomatitis (RAS). Each radial segment represents an individual taxon, organized by taxonomic hierarchy (phylum, class, order, family, genus, and species) along the outer arc. Concentric rings display p-values from multiple MR analytical methods, including inverse-variance weighted (IVW), MR-Egger, weighted median, MR-Egger intercept, MR-PRESSO global test, and Cochran’s Q heterogeneity test. Color gradient ranges from deep red (P approaching 0) through white (p=0.05) to purple (p=1.0), with red shading indicating statistical significance. The innermost ring shows odds ratios (OR), where orange dots denote risk factors (OR > 1) and green dots denote protective factors (OR < 1).

Our UVMR investigation into whether perturbations in oral microbial communities contribute causally to RAS development revealed a single bacterial taxon with significant protective properties. The Order Clostridiales demonstrated a reduced likelihood of RAS occurrence (OR = 0.851, 95% CI: 0.759–0.954, p=0.006), as illustrated in Fig 1 and detailed in Table A2. Complementary analytical approaches, including weighted median and MR-Egger methods, yielded concordant effect estimates (Table A2). When examining potential reverse causality through bidirectional MR, no evidence emerged for RAS influencing microbial abundance (Table A3). Furthermore, diagnostic assessments using MR-PRESSO failed to identify outlier variants (p>0.05), while the MR-Egger intercept test indicated the absence of horizontal pleiotropy (p>0.05; Table A4). Collectively, these results support the Order Clostridiales as a protective factor against RAS development.

### Metabolomic Determinants of RAS Susceptibility

**Fig 2:**
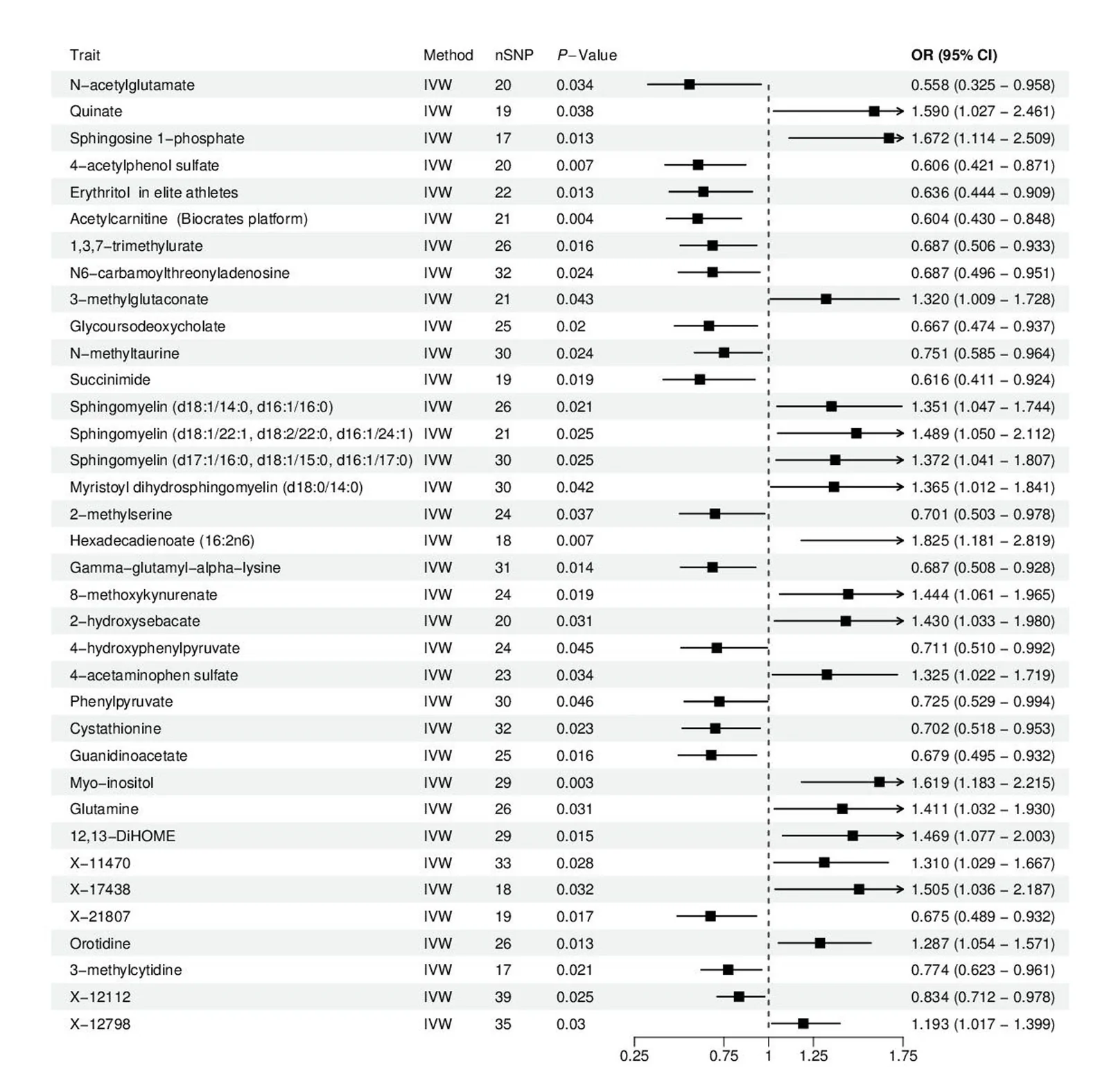
Causal associations between blood metabolites and RAS using two-sample Mendelian randomization. Forest plot of the causal associations between blood metabolites and recurrent aphthous stomatitis (RAS) identified by two-sample Mendelian randomization. Odds ratios (OR) and 95% confidence intervals (95% CI) were estimated using the inverse-variance weighted (IVW) method. Black squares represent point estimates of OR, and horizontal lines indicate 95% CI. The vertical dashed line at OR = 1.0 represents the null hypothesis of no causal effect. Metabolites with OR > 1 (right of the dashed line) are associated with increased RAS risk, while those with OR < 1 (left) are associated with reduced risk. Only metabolites reaching statistical significance (p<0.05) are displayed. nSNP, number of single nucleotide polymorphisms used as instrumental variables.

The systematic evaluation of metabolite-RAS relationships through IVW analysis uncovered 36 metabolites exhibiting statistically significant causal associations (Fig 2; Table A5). These compounds represent multiple metabolic pathways, indicating widespread metabolic involvement in RAS etiology. Among risk-increasing metabolites, myo-inositol displayed the most statistically significant association (OR = 1.619, 95% CI: 1.183–2.215, p=0.003), followed by hexadecadienoate (16:2n-6) (OR = 1.825, 95% CI: 1.181–2.819, p=0.007) and sphingosine 1-phosphate (OR = 1.672, 95% CI: 1.114–2.509, p=0.013). Conversely, several metabolites conferred protection, including 4-acetylphenol sulfate (OR = 0.606, 95% CI: 0.421–0.871, p=0.007), acetylcarnitine (OR = 0.604, 95% CI: 0.430–0.848, p=0.004), and erythritol (OR = 0.636, 95% CI: 0.444–0.909, p=0.013). Sensitivity analyses employing weighted median and MR-Egger approaches reinforced these observations (Supplementary Table A5). Neither the MR-Egger intercept test (p>0.05) nor MR-PRESSO global test (p>0.05) suggested confounding by pleiotropy (Table A6), confirming the validity of the results.

### Inflammatory Protein Associations with RAS

Investigation of circulating inflammatory mediators as potential RAS risk factors yielded null findings. Despite comprehensive MR evaluation of multiple inflammatory proteins, none achieved statistical significance for causal associations with RAS susceptibility (Table A7). Robustness checks through MR-Egger and MR-PRESSO methods supported the absence of meaningful relationships (p>0.05; Table A8).

### Mediation Pathway Analysis

**Fig 3:**
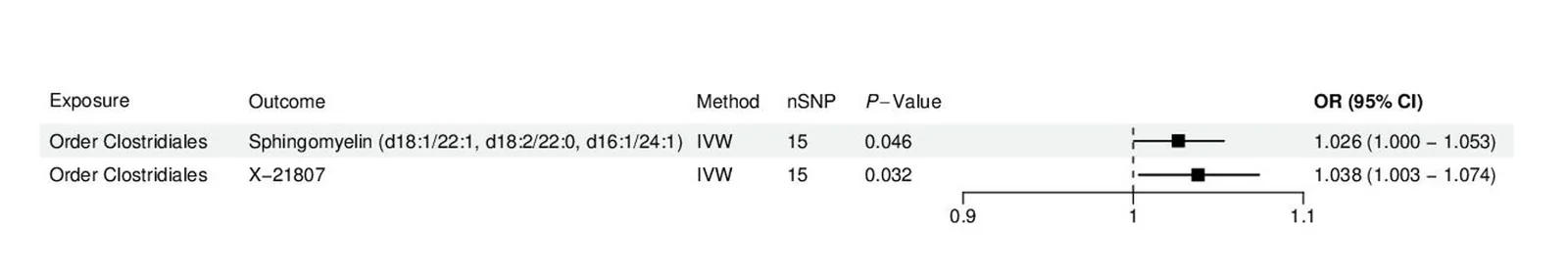
Causal associations between oral microbiota and blood metabolites using two-sample Mendelian randomization. Forest plot of the causal associations between oral microbiota and blood metabolites identified by two-sample Mendelian randomization. Odds ratios (OR) and 95% confidence intervals (95% CI) were estimated using the inverse-variance weighted (IVW) method. This analysis represents the first step of the two-step mediation MR framework, identifying circulating metabolites that are causally influenced by oral microbial taxa. Black squares represent point estimates of OR, and horizontal lines indicate 95% CI. The vertical dashed line at OR = 1.0 represents the null hypothesis. Only metabolites reaching statistical significance (p<0.05) are displayed. nSNP, number of single nucleotide polymorphisms used as instrumental variables.

To elucidate potential mechanistic links connecting oral microbiota with systemic metabolism in RAS pathogenesis, we implemented two-step MR analyses. Initial screening identified metabolites influenced by the Order Clostridiales, with notable increases in sphingomyelin (d18:1/22:1, d18:2/22:0, d16:1/24:1) levels (OR = 1.026, 95% CI: 1.000–1.053, p=0.046) and X-21807 abundance (OR = 1.038, 95% CI: 1.003–1.074, p=0.032) (Fig 3; Table A9). Subsequent MVMR modeling quantified the independent contributions of Clostridiales and sphingomyelin to RAS risk. The analysis demonstrated that the Clostridiales-induced elevation of sphingomyelin (d18:1/22:1, d18:2/22:0, d16:1/24:1) contributed to heightened RAS susceptibility (OR = 1.627, 95% CI: 1.092–2.424, p=0.017). However, after accounting for sphingomyelin concentrations, the Order Clostridiales maintained its beneficial influence on RAS prevention (OR = 0.837, 95% CI: 0.747–0.939, p=0.002; Table 1). These results suggest that the protective effect of Clostridiales on RAS is partially counteracted through metabolic intermediates.

## DISCUSSION

This study employed a multi-layered MR framework to investigate causal relationships between oral microbiota, circulating metabolites, inflammatory proteins, and RAS, revealing a complex microbiome–metabolome–disease axis that may underlie oral mucosal disease susceptibility. Through UVMR and MVMR analyses, we identified the Order Clostridiales as a statistically significant protective factor against RAS, while multiple lipid mediators including sphingosine-1-phosphate (S1P), three sphingomyelin species, and hexadecadienoate emerged as risk factors, alongside protective metabolites spanning antioxidant defense and energy metabolism pathways. Most notably, our two-step mediation MR analysis identified sphingomyelin (d18:1/22:1, d18:2/22:0, d16:1/24:1) as a key metabolic intermediate linking Clostridiales to RAS susceptibility, with the direct protective effect of Clostridiales persisting after adjustment for sphingomyelin levels. These findings provide genetic evidence supporting potential mechanistic links in RAS pathogenesis and suggest candidate targets — including Clostridiales-based probiotic supplementation and sphingomyelin pathway modulation — that warrant further experimental validation. By leveraging genetic instruments to reduce confounding and reverse causation bias, the present MR analysis provides genetic evidence that may inform future translational research linking microbiome-metabolome interactions to oral mucosal diseases.

The Order Clostridiales emerged as the only microbial taxon with a statistically significant protective effects against RAS, providing genetic evidence for the microbial regulation of oral mucosal immune homeostasis. The biological basis of this protective effect is primarily attributable to its robust butyrate-producing capacity. Butyrate-producing bacteria within the Clostridiales order not only provide energy for mucosal epithelium but also regulate epithelial inflammation and immune tolerance by inducing anti-inflammatory cytokines and tolerogenic dendritic cells, thereby reinforcing the barrier function in various mucosal disease models.^37^ At the molecular level, butyrate reduces epithelial permeability by upregulating tight junction proteins and enhancing transepithelial electrical resistance, thereby protecting mucosal barrier integrity under oxidative stress conditions.^27^ Similarly, *Clostridium butyricum* and its derived extracellular vesicles strengthen epithelial tight junctions, suppress pro-inflammatory cytokine release, and reverse barrier disruption in inflammatory mucosal disease models.^30^ Beyond the butyrate pathway, the 7α-dehydroxylating strain *Clostridium scindens* within Clostridiales accelerates mucosal healing in injury models by restoring secondary bile-acid homeostasis and driving epithelial regeneration,^20^ suggesting that members of this order possess multiple protective mechanisms. At the immune regulatory level, the core mechanism underlying the Clostridiales–RAS protective association involves the induction of regulatory T-cells (Treg). Co-transplantation of commensal bacteria from Clostridiales alleviates allergic inflammation through MyD88-dependent RORγt+ Treg induction pathways.^1^ Notably, novel Clostridiales strains isolated from human populations potently induce colonic RORγt+ Treg differentiation in germ-free mice when colonized as single strains,^39^ demonstrating the central role of this bacterial order in maintaining mucosal tolerance through immune regulation. Consistent with these experimental findings, clinical studies have shown that the proportion and function of CD4+CD25+FOXP3+ Treg in peripheral blood are significantly impaired in RAS patients,^25^ suggesting that Treg deficiency may weaken mucosal immune tolerance and increase recurrence risk. Supporting the clinical relevance of these observations, a prospective cohort study found that head-and-neck–cancer patients receiving radiotherapy who had received a pre-treatment fecal enrichment of *Faecalibacterium* (a representative genus of the butyrate-producing Clostridiales) showed a significantly reduced risk of radiation-related oral mucositis and tumor recurrence.^2^ In summary, our MR analysis provided genetic evidence supporting a causal link between Clostridiales and reduced RAS risk, which is biologically supported by converging evidence from butyrate-mediated barrier protection, Treg induction, and bile-acid homeostasis. These findings suggest new therapeutic avenues for RAS management based on probiotic supplementation or butyrate administration.

In contrast to the protective role of Clostridiales, our analysis identified multiple lipid signaling molecules that increase RAS susceptibility through pro-inflammatory mechanisms, with S1P, sphingomyelin (d18:1/22:1, d18:2/22:0, d16:1/24:1), hexadecadienoate, and 12,13-DiHOME showing the most prominent effects. S1P operates through its steep concentration gradient formed between blood-lymph and tissues, driving T-cell egress from secondary lymphoid organs and compromising vascular integrity, thereby accelerating inflammatory cell aggregation at damaged mucosal sites and potentially exacerbating local immune responses in oral ulceration.^32^ This pro-inflammatory amplification through lymphocyte trafficking and innate-adaptive immune coupling has been demonstrated in various autoimmune and inflammatory diseases. The clinical efficacy of fingolimod, an S1P receptor modulator, in multiple sclerosis treatment further supports this mechanism.^49^ Through a distinct pathway, excess membrane sphingomyelins promote inflammation via acid sphingomyelinase-mediated hydrolysis to ceramide, which disrupts cell membrane microdomain stability and amplifies inflammatory signaling cascades in mucosal tissues.^21^ Ceramide accumulation further induces intestinal epithelial cell apoptosis and weakens mucosal barrier function, facilitating inflammatory factor penetration and exacerbating tissue destruction in ulcerative lesions.^11^ Building upon this compromised barrier, the ω-6 polyunsaturated hexadecadienoate (16:2n-6) has been identified as an elevated metabolite in chronic inflammatory states associated with smoking, and may amplify inflammatory responses through the pathway from lipid peroxidation to NF-κB activation.^15^ Similarly, 12,13-DiHOME — an epoxide hydrolysis product of linoleic acid — modulates neutrophil respiratory burst and reshapes innate immune responses in acute and chronic inflammation models.^19^ Collectively, these lipid mediators form a pro-inflammatory network that promotes RAS development through synergistic activation of inflammatory signaling, disruption of mucosal barrier integrity, and alteration of immune cell trafficking.

Beyond this lipid–driven inflammatory network, our MR analysis also identified a separate class of risk-associated small-molecule organic acids and their derivatives — including myo-inositol, quinate, orotidine, 3-methylglutaconate, and 8-methoxykynurenate — that contribute to RAS pathophysiology through metabolic mechanisms independent of lipid signaling. Myo-inositol promotes NLRP3 inflammasome stabilization and amplifies inflammatory cascades in neural and myocardial tissues through its rate-limiting metabolic enzyme MIOX, suggesting that dysregulation of this metabolite may serve as an upstream driver linking inflammation susceptibility to barrier disruption.^54^ Quinate, a key intermediate in the shikimate pathway of aromatic acid metabolism, has been reported to modulate the DR3/IKK/NF-κB signaling axis in animal models.^26^ While acute quinate administration attenuates neuroinflammation experimentally, genetically determined chronic elevation of circulating quinate may reflect sustained perturbation of aromatic acid flux, potentially disrupting microbial-host metabolic crosstalk and shifting the mucosal immune environment toward inflammatory susceptibility. At the level of energy metabolism, pyrimidine pathway disturbance contributes through an altered nucleotide supply. Orotidine is converted from orotate in the de novo pyrimidine synthesis pathway. Experimental studies on its upstream precursor orotate have shown that supplementation statistically significantly increases small intestinal villus height, crypt depth, and DNA content in animal models,^10^ supporting the role of this metabolic axis in driving mucosal cell proliferation through accelerated nucleotide availability. In RAS, systemic orotidine elevation may create an imbalance between proliferation and energy consumption, where excessive cell turnover depletes energy reserves, paradoxically prolonging ulcer cycles. This proliferation-energy imbalance hypothesis is supported by the finding of 3-methylglutaconate, a recognized marker of mitochondrial dysfunction that amplifies energy crisis and drives cellular stress in atypical 3-methylglutaconic aciduria.^50^ Extending this connection between energy crisis and inflammation, the tryptophan-to-kynurenine pathway derivative 8-methoxykynurenate also demonstrated risk-increasing effects. Although no studies have directly examined 8-methoxykynurenate, prospective population studies of its parent compound kynurenate demonstrate that elevated circulating levels correlate positively with metabolic dysregulation and type 2 diabetes risk,^35^ suggesting that perturbations along this metabolic branch may amplify inflammation through immunometabolic pathways. Taken together, the synergistic disturbance of these metabolic pathways constitutes a pro-inflammatory network centered on the interplay of energy imbalance, oxidative stress, and immune activation.

Conversely, the present MR analysis identified four metabolites with statistically significant protective effects — 4-acetylphenol sulfate, erythritol, 4-hydroxyphenylpyruvate (HPP), and phenylpyruvate — that operate through detoxification, antioxidant defense, and aromatic amino-acid catabolism, suggesting endogenous compensatory mechanisms against RAS. As a representative detoxification metabolite, 4-acetylphenol sulfate achieves phenol sequestration through sulfation. Algae model studies indicate that phenolic compounds statistically significantly reduce toxicity after sulfation,^24^ a mechanism that may maintain epithelial integrity by neutralizing toxic phenolic substances in the oral mucosal environment. Erythritol, a natural four-carbon polyol, exhibits multiple protective effects including ABTS/DPPH radical scavenging and significant elevation of glutathione and superoxide dismutase levels in diabetic rat models,^45^ properties that are relevant to maintaining oral mucosal redox balance. Among aromatic amino-acid metabolites, HPP inhibits excessive ROS generation and reduces mucous cell apoptosis through activation of its downstream enzyme HPPD in sea cucumber models,^28^ while phenylpyruvate and its glucoside derivatives independently suppress high glucose-induced ROS accumulation and exert anti-apoptotic effects in cardiomyocyte models.^9^ Collectively, detoxification sulfation, free-radical scavenging, and aromatic amino-acid metabolism form an antioxidant and barrier defense axis, suggesting that these protective metabolites synergistically enhance mucosal antioxidant capacity to attenuate RAS pathological progression. A separate cluster of seven protective metabolites operates through energy and amino acid metabolism to support mucosal repair, primarily through mitochondrial energy metabolism, glutamate–glutathione cycling, the transsulfuration pathway, and one-carbon metabolism. On the mitochondrial energy axis, acetylcarnitine maintains the connection between β-oxidation and the TCA cycle by transporting acetyl groups across mitochondrial membranes.^8^ Succinamide and its pharmacological analogs improve energy crisis and hepatorenal dysfunction after tissue hypoxia through TCA intermediate supplementation in animal models, functioning as a rapidly reversible mitochondrial fuel buffer.^52^ Guanidinoacetate, as a phosphocreatine precursor, increases brain and peripheral creatine stores in humans, thereby replenishing ATP and stabilizing cellular energy homeostasis under ischemic conditions.^33^ Within the amino acid regulatory network, N-acetylglutamate — an essential cofactor for the urea cycle rate-limiting enzyme CPS1 — drives ammonia-to-urea conversion while providing precursors for the glutamate–glutathione axis, thereby coupling ammonia clearance, antioxidant defense, and energy redistribution.^42^ The transsulfuration intermediate cystathionine diverts homocysteine to cysteine production, supplying reductive sulfur sources for glutathione and hydrogen sulfide to buffer inflammatory oxidative pressure.^40^ Additionally, 2-methylserine participates in one-carbon metabolism, supporting nucleotide synthesis and providing methylation substrates for mucosal repair. At the structural level, γ-glutamyl-α-lysine — an ε-(γ-glutamyl)-lysine crosslink product catalyzed by transglutaminase — stabilizes extracellular matrix and epithelial structure, serving as a molecular marker of tissue repair activation.^14^ This structural reinforcement synergizes with the energy and amino acid regulatory mechanisms to support mucosal barrier reconstruction.

The metabolites identified above do not act independently of the oral microbiome. Our two-step mediation MR analysis revealed how oral microbiota influence RAS susceptibility by modulating specific metabolic products, most notably through the Clostridiales–sphingomyelin–RAS causal chain. Although observational multi-omics studies have reported associations between Clostridiales members and host sphingolipid metabolism, the directionality of these relationships has varied across study designs and populations.^29,43^ Our MR analysis, which leverages genetic instruments to minimize confounding and reverse causation, provides complementary causal evidence linking Clostridiales abundance to sphingomyelin levels, with elevated sphingomyelin in turn increasing RAS susceptibility. The pathogenic consequences of sphingomyelin accumulation have been characterized in animal models, where dietary or endogenous sphingomyelin accumulation exacerbates mucosal inflammation and ulcer formation through epithelial cathepsin D-mediated apoptosis.^11^ Based on this understanding, pharmacological strategies targeting the sphingomyelin pathway have shown promise. Acid sphingomyelinase inhibition attenuates mucosal inflammation and reduces ulcerative lesions in murine models,^36^ and amitriptyline mouthwash — which possesses acid sphingomyelinase inhibitory activity — has been reported to relieve oral mucositis pain in head and neck cancer patients receiving chemoradiotherapy,^22^ suggesting feasibility of targeting this pathway in oral mucosal disease management. Overall, the present MR mediation analysis provided genetic evidence in support of a potential causal pathway from Clostridiales through sphingomyelin to RAS, suggesting candidate targets for oral mucosal disease intervention through the microbe-metabolite axis. Future prospective clinical trials in RAS patients are needed to validate the clinical efficacy of Clostridiales probiotic supplementation or sphingomyelin pathway inhibition for RAS prevention and treatment.

Several limitations should be acknowledged. First, the GWAS datasets used in this analysis were predominantly derived from populations of European ancestry, which may limit the generalizability of our findings to non-European populations. Second, the oral microbiome GWAS had a relatively modest sample size, which may have limited statistical power for detecting causal associations with smaller effect sizes. Third, a relaxed significance threshold of p<5 × 10⁻⁵ was adopted for instrumental variable selection to ensure sufficient instrument numbers, which may increase susceptibility to weak-instrument bias despite F-statistic filtering.

## CONCLUSION

This two-sample Mendelian randomization study identified genetic evidence supporting a protective association between the order Clostridiales and recurrent aphthous stomatitis, together with risk-increasing associations for several circulating metabolites, including sphingomyelin species. The protective association of Clostridiales remained statistically significant after adjustment for sphingomyelin, whereas the Clostridiales-associated increase in sphingomyelin was associated with higher RAS risk, indicating that sphingomyelin may partially counteract the protective association between Clostridiales and RAS. These findings prioritize the oral microbiome–metabolite axis for experimental validation but do not establish the clinical efficacy of probiotic supplementation or sphingomyelin-targeted treatment.

## ACKNOWLEDGEMENTS 

We gratefully acknowledge the original authors for providing the datasets used in this research. This study was supported by the Natural Science Foundation of Hunan Province of China (Grant No. 2025JJ80945) and the Scientific Research Fund of Hunan Provincial Education Department (Grant No. 25B0336).
